# Low-Value Prostate-Specific Antigen Screening in Older Males

**DOI:** 10.1001/jamanetworkopen.2023.7504

**Published:** 2023-04-11

**Authors:** Sandhya Kalavacherla, Paul Riviere, Juan Javier-DesLoges, Matthew P. Banegas, Rana R. McKay, James D. Murphy, Brent S. Rose

**Affiliations:** 1 University of California San Diego School of Medicine, La Jolla; 2 Department of Radiation Medicine and Applied Sciences, University of California San Diego, La Jolla; 3 Department of Urology, University of California San Diego, La Jolla; 4 Division of Hematology-Oncology, University of California San Diego, La Jolla

## Abstract

**Importance:**

The US Preventive Services Task Force guidelines advise against prostate-specific antigen (PSA) screening for prostate cancer in males older than 69 years due to the risk of false-positive results and overdiagnosis of indolent disease. However, this low-value PSA screening in males aged 70 years or older remains common.

**Objective:**

To characterize the factors associated with low-value PSA screening in males 70 years or older.

**Design, Setting, and Participants:**

This survey study used data from the 2020 Behavioral Risk Factor Surveillance System (BRFSS), a nationwide annual survey conducted by the Centers for Disease Control and Prevention that collects information via telephone from more than 400 000 US adults on behavioral risk factors, chronic illnesses, and use of preventive services. The final cohort comprised male respondents to the 2020 BRFSS survey who were categorized into the following age groups: 70 to 74 years, 75 to 79 years, or 80 years or older. Males with a former or current prostate cancer diagnosis were excluded.

**Main Outcomes and Measures:**

The outcomes were recent PSA screening rates and factors associated with low-value PSA screening. Recent screening was defined as PSA testing within the past 2 years. Weighted multivariable logistic regressions and 2-sided significance tests were used to characterize factors associated with recent screening.

**Results:**

The cohort included 32 306 males. Most of these males (87.6%) were White individuals, whereas 1.1% were American Indian, 1.2% were Asian, 4.3% were Black, and 3.4% were Hispanic individuals. Within this cohort, 42.8% of respondents were aged 70 to 74 years, 28.4% were aged 75 to 79 years, and 28.9% were 80 years or older. The recent PSA screening rates were 55.3% for males in the 70-to-74-year age group, 52.1% in the 75-to-79-year age group, and 39.4% in the 80-year-or-older group. Among all racial groups, non-Hispanic White males had the highest screening rate (50.7%), and non-Hispanic American Indian males had the lowest screening rate (32.0%). Screening increased with higher educational level and annual income. Married respondents were screened more than unmarried males. In a multivariable regression model, discussing PSA testing advantages with a clinician (odds ratio [OR], 9.09; 95% CI, 7.60-11.40; *P* < .001) was associated with increased recent screening, whereas discussing PSA testing disadvantages had no association with screening (OR, 0.95; 95% CI, 0.77-1.17; *P* = .60). Other factors associated with a higher screening rate included having a primary care physician, a post–high school educational level, and income of more than $25 000 per year.

**Conclusions and Relevance:**

Results of this survey study suggest that older male respondents to the 2020 BRFSS survey were overscreened for prostate cancer despite the age cutoff for PSA screening recommended in national guidelines. Discussing the benefits of PSA testing with a clinician was associated with increased screening, underscoring the potential of clinician-level interventions to reduce overscreening in older males.

## Key Points

**Question:**

What factors are associated with low-value prostate-specific antigen (PSA) screening among older males?

**Findings:**

In this survey study of 32 306 male respondents aged 70 years or older, the overall PSA screening rate was approximately 50%. Clinician-led discussion of PSA testing advantages was associated with increased screening, whereas PSA testing disadvantage discussion had no association with screening.

**Meaning:**

Findings of this study suggest that clinician-level interventions have the potential to reduce overscreening for prostate cancer in older males.

## Teaser

This survey study examines the pattern of and factors associated with prostate cancer screening in US males aged 70 years or older.

## Introduction

Prostate cancer is the fifth leading cause of cancer-related deaths in males worldwide.^1^ While screening for prostate cancer may reduce prostate cancer–specific mortality, it comes at the risk of overdiagnosis, particularly in older males or those with comorbid medical conditions.^2,3^ Screening with serum prostate-specific antigen (PSA) in males who are unlikely to benefit from intervention for prostate cancer exposes these individuals to anxiety, invasive biopsies, and possible overtreatment, which could lead to unnecessary clinical consequences.^4,5^

Given the lack of evidence of clinical benefit of PSA screening for prostate cancer in older males, the US Preventive Services Task Force (USPSTF) published revised guidelines in 2018 that advised against PSA screening for prostate cancer in males 70 years or older due to the risk of false-positive results and overdiagnosis of indolent disease.^6^ These guidelines now recommend that males aged 55 to 69 years make the personal decision to undergo PSA screening after discussing its potential benefits and risks with their physician.^6^

To characterize the factors associated with low-value PSA screening in males 70 years or older, we analyzed a cohort of males from the Behavioral Risk Factor Surveillance System (BRFSS), a nationwide survey conducted by the Centers for Disease Control and Prevention. We hypothesized that, despite the advice to the contrary, PSA screening in older males remains overused.

## Methods

Every year, the BRFSS surveys a random sample of more than 400 000 adults in the US via telephone on their behavioral risk factors, chronic illnesses, and use of preventive services such as PSA screening. The BRFSS survey response data are published online by the Centers for Disease Control and Prevention along with weights that allow for any conclusions derived from this survey cohort to be better representative of the national population.^7,8^ The University of California San Diego Institutional Review Board deemed this survey study exempt from review and the informed consent requirement because the data were published by a federal agency, publicly available, and deidentified. We followed the American Association for Public Opinion Research (AAPOR) reporting guideline.

We analyzed the 2020 BRFSS survey data on the PSA-related questions. These questions and possible responses were as follows: Have you ever had a PSA test? (response options: yes or no); How long has it been since you had your PSA test? (response options: within the past year, 1-2 years ago, 2-3 years ago, 3-5 years ago, or more than 5 years ago [upper bounds were noninclusive]); Has a doctor, nurse, or other health professional ever talked with you about the benefits of the PSA test? (response options: yes or no); and Has a doctor, nurse, or other health professional ever talked with you about the harms of the PSA test? (response options: yes or no). Respondents could select *don’t know/not sure* for any of these questions. If a respondent refused to answer a question, the item was marked as *refused* or *blank* within the survey database, and these response types were recoded as *NA* (not available).^9^

We also collected from the BRFSS survey data the demographic characteristics of age, sex, race and ethnicity (Hispanic, Non-Hispanic Asian [hereafter Asian], Non-Hispanic Black [hereafter Black], Non-Hispanic American Indian [hereafter American Indian], or Non-Hispanic White [hereafter White]), marital status (never married; married; divorced, widowed, or separated; or unmarried partnership), annual income (<$25 000, $25 000-$50 000, >$50 000-$74 000, >$75 000), educational level (no high school diploma or attendance, high school diploma or GED [General Educational Development] certificate, some college, or college degree), employment status (employed, unemployed, homemaker, student, or retired), and smoking status (never smoker, every day, or some days); prostate cancer diagnosis history; and access-to-care information, including whether respondents had a primary care physician (PCP; yes or no) and any cost barrier to care (yes or no). The associations of these variables with recent PSA screening within the study cohort were specifically assessed, as these variables have been reported as factors in PSA screening use in target populations.^10,11,12,13,14,15,16,17,18^

Based on their responses regarding receipt of a PSA test and time elapsed since their last PSA test, the male respondents were grouped into mutually exclusive, time-based PSA screening categories: never, past year, past 2 years, past 3 years, past 5 years, or more than 5 years. Given the national PSA screening guidelines, respondents were considered to be *recently screened* if they had a PSA test within the past 2 years. Furthermore, since we focused this study on PSA screening in males who were older than the recommended screening age range, we limited the data to a subset of males who were 70 years or older. Respondent ages within this cohort were organized into the following age groups: 70 to 74 years, 75 to 79 years, 80 years or older (inclusive bounds). To avoid conflating PSA screening with PSA testing for monitoring of a known prostate cancer diagnosis, we excluded from the study males who had a former or current prostate cancer diagnosis.

### Statistical Analysis

All data analyses were performed with R, version 4.1.3 (R Foundation for Statistical Computing), using core functions, the haven package for data import and manipulation, and the survey package for weighted logistic regression analyses.^19,20,21^ Respondent demographic characteristics were compared among the 3 age groups using the Pearson χ^2^ tests. Recent PSA screening rates based on demographic characteristics were calculated according to American Association for Public Opinion Research guidelines and compared using analysis of variance tests.^9^ Weighted univariable logistic regressions were performed to assess the association of recent PSA screening with each of the following variables: age group, race and ethnicity, annual income, educational level, employment status, marital status, smoking status, having a cost barrier to care, having a PCP, and having discussions with a clinician about the advantages and/or disadvantages of PSA testing. A weighted multivariable logistic regression model was constructed to measure the outcomes of the variables that were independently associated with recent screening in the univariable regressions. The results from regression models were presented as odds ratios (ORs) with 95% CIs. For all statistical tests, a 2-sided *P* < .05 was considered to be significant. In a sensitivity analysis, we repeated the key analyses using unweighted survey data.

## Results

### Cohort Selection

**Figure 1.**
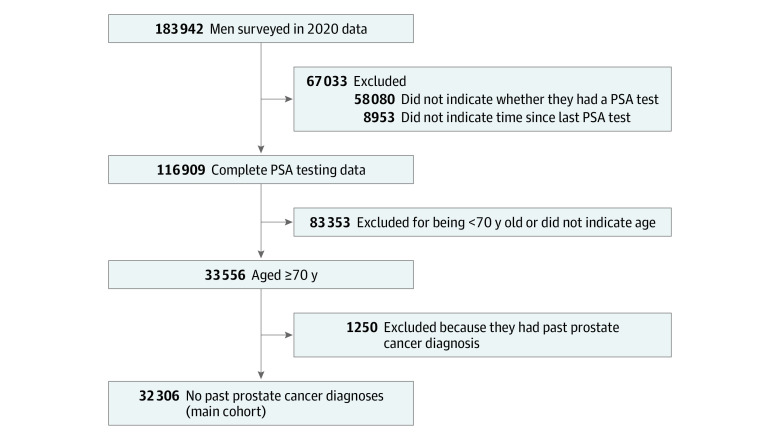
Final Cohort Selection Flowchart PSA indicates prostate-specific antigen.

The final cohort consisted of 32 306 male respondents (Figure 1). Most of these males (87.6%) were White individuals, whereas 1.1% were American Indian, 1.2% were Asian, 4.3% were Black, and 3.4% were Hispanic individuals. Within this cohort, 42.8% of respondents were aged 70 to 74 years, 28.4% were aged 75 to 79 years, and 28.9% were 80 years or older (Table 1). White race and ethnicity was the most represented in all 3 age groups.

**Table 1.** Male Respondent Characteristics

Characteristic	Respondents, No. (%)			*P* value^a^
	Aged 70-74 y (n = 13 811)	Aged 75-79 y (n = 9165)	Aged ≥80 y (n = 9330)	
Race and ethnicity^b^				
American Indian	178 (1.3)	110 (1.2)	71 (0.8)	<.001
Asian	188 (1.4)	108 (1.2)	94 (1.0)	
Black	653 (4.7)	370 (4.0)	382 (4.1)	
Hispanic	523 (3.8)	313 (3.4)	258 (2.8)	
White	11 927 (86.0)	8075 (88.0)	8319 (89.0)	
Unknown	342 (2.5)	189 (2.1)	206 (2.2)	
Annual income, $^c^				
<25 000	2104 (15.0)	1462 (16.0)	1818 (19.0)	<.001
25 000-50 000	3140 (23.0)	2169 (24.0)	2529 (27.0)	
>50 000-74 000	2256 (16.0)	1502 (16.0)	1301 (14.0)	
>75 000	4150 (30.0)	2525 (28.0)	1937 (21.0)	
Unknown	2161 (16.0)	1507 (16.0)	1745 (19.0)	
Educational level				
No high school diploma or attendance	669 (4.8)	652 (7.1)	772 (8.3)	<.001
High school diploma or GED certificate	2936 (21.0)	2232 (24.0)	2608 (28.0)	
Some college	3763 (27.0)	2122 (23.0)	1989 (21.0)	
College degree	6418 (46.0)	4139 (45.0)	3930 (42.0)	
Unknown	25 (0.2)	20 (0.2)	31 (0.3)	
Marital status				
Never married	908 (6.6)	422 (4.6)	314 (3.4)	<.001
Married	9202 (67.0)	5968 (65.0)	5047 (54.0)	
Divorced, widowed, or separated	3471 (25.0)	2642 (29.0)	3873 (42.0)	
Unmarried partnership	192 (1.4)	106 (1.2)	59 (0.6)	
Unknown	38 (0.3)	27 (0.2)	37 (0.4)	
Smoking status				
Never smoker	6312 (83.0)	4729 (88.0)	4880 (94.0)	<.001
Every day	988 (13.0)	502 (9.3)	240 (4.6)	
Some days	320 (4.0)	150 (2.8)	88 (1.7)	
Employment status				
Employed	2735 (20.0)	1293 (14.0)	715 (7.7)	<.001
Unemployed	596 (4.3)	295 (3.2)	227 (2.4)	
Homemaker	8 (<0.1)	5 (<0.1)	16 (0.2)	
Student	15 (0.1)	8 (<0.1)	5 (<0.1)	
Retired	10 407 (75.0)	7539 (82.0)	8322 (89.0)	
Unknown	50 (0.3)	25 (0.2)	45 (0.4)	
Cost barrier to care				
Yes	450 (3.3)	300 (3.3)	315 (3.4)	.90
No	13 332 (97.0)	8844 (97.0)	8973 (97.0)	
Have a PCP				
Yes	12 636 (91.0)	8560 (93.0)	8628 (92.0)	<.001
No	1125 (8.1)	568 (6.2)	646 (6.9)	
Unknown	50 (0.3)	37 (0.2)	56 (0.6)	
Time since last PSA test^d^				
Never	4003 (29.0)	2624 (29.0)	3191 (34.0)	<.001
Past y	6174 (45.0)	3879 (42.0)	2910 (31.0)	
1-2 y ago	1461 (11.0)	900 (9.8)	762 (8.2)	
2-3 y ago	658 (4.8)	479 (5.2)	455 (4.9)	
3-5 y ago	596 (4.3)	462 (5.0)	527 (5.6)	
>5 y ago	919 (6.7)	821 (9.0)	1485 (16.0)	

Abbreviations: GED, General Educational Development; PCP, primary care physician; PSA, prostate-specific antigen.

^a^ Calculated using Pearson χ^2^ test.

^b^ Race and ethnicity were collected from the 2020 Behavioral Risk Factor Surveillance System survey data.

^c^ Estimated by zip code; lower bounds were noninclusive.

^d^ Upper bounds were noninclusive.

Most males in each age group were retired, had a college degree, and were never smokers. More males in the 70-to-74-year and 75-to-79-year age groups had an annual income of more than $50 000 compared with those in the 80-year-or-older group, whereas more respondents 80 years or older were divorced, widowed, or separated compared with those aged 70 to 79 years. Males in the 80-year-or-older group were more likely to be last screened more than 5 years ago compared with those in the 70-to-74-year and 75-to-79-year age groups (16.0% vs 6.7% and 9.0%, respectively; *P* < .001) and were less likely to be last screened within the past year as compared with those aged 70 to 74 years or 75 to 79 years (31.0% vs 45.0% and 42.0%, respectively; *P* < .001).

### PSA Screening and Age

**Figure 2.**
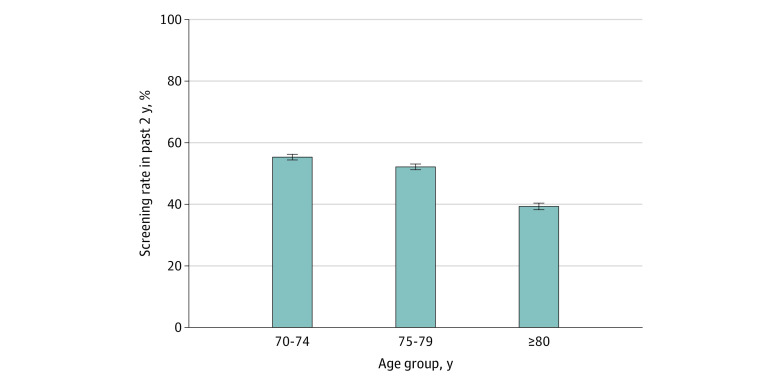
Screening Rates by Age Group Error bars represent 95% CIs.

The recent screening rates were 55.3% (95% CI, 54.0%-56.5%) for males in the 70-to-74-year age group, 52.1% (95% CI, 50.7%-53.6%) in the 75-to-79-year age group, and 39.4% (95% CI, 38.1%-40.6%) in the 80-year-or-older group (Figure 2), and the differences were significant at *P* < .001. Among the full cohort, 81.3% (95% CI, 78.2%-85.1%) of respondents were screened within the past year. In a multivariable regression model, compared with respondents aged 70 to 74 years, those who were 80 years or older were less likely to be screened (OR, 0.54; 95% CI, 0.44-0.66; *P* < .001), whereas those aged 75 to 79 years were not more or less likely to be recently screened (OR, 0.86; 95% CI, 0.71-1.04; *P* = .13). Factors associated with increased screening in older males included having a PCP (OR, 1.89; 95% CI, 1.41-2.53; *P* < .001) and discussing the advantages of PSA testing with a clinician (OR, 9.09; 95% CI, 7.60-11.40; *P* < .001) (Table 2). Discussing the disadvantages of PSA testing with a clinician had no association with recent screening in this population (OR, 0.95; 95% CI, 0.77-1.17; *P* = .60). Because the discussion of PSA testing advantages with a clinician was associated with recent screening, we studied the interaction between respondent age and discussion of PSA testing advantages and disadvantages with a clinician. A total of 8376 males (25.9%) reported discussing both PSA testing advantages and disadvantages with a clinician; the correlation coefficient between discussion of advantages and discussion of disadvantages was *R* = 0.42 (95% CI, 0.41-0.42; *P* < .001).

**Table 2.** Multivariable Logistic Regression Assessing Odds of Recent PSA Screening

Characteristic	Univariate OR (95% CI)	*P* value	Multivariate OR (95% CI)	*P* value
Age group, y				
70-74	1 [Reference]		1 [Reference]	
75-79	0.86 (0.74-0.98)	.03	0.86 (0.71-1.04)	.13
≥80	0.54 (0.48-0.62)	<.001	0.54 (0.44-0.66)	<.001
Have a PCP				
No	1 [Reference]		1 [Reference]	
Yes	3.22 (2.63-4)	<.001	1.89 (1.41-2.53)	<.001
Discussed PSA testing advantages with a clinician				
No	1 [Reference]		1 [Reference]	
Yes	10 (9.09-12.5)	<.001	9.09 (7.6-11.4)	<.001
Discussed PSA testing disadvantages with a clinician				
No	1 [Reference]		1 [Reference]	
Yes	2.56 (2.27-2.94)	<.001	0.95 (0.77-1.17)	.60
Race and ethnicity^a^				
American Indian	0.51 (0.31-0.85)	.01	0.58 (0.32-1.06)	.08
Asian	1.12 (0.58-2.15)	.70	0.71 (0.34-1.49)	.40
Black	0.76 (0.61-0.95)	.02	0.87 (0.64-1.19)	.40
Hispanic	0.62 (0.45-0.84)	.002	1.28 (0.82-2.01)	.30
White	1 [Reference]		1 [Reference]	
Annual income, $^b^				
<25 000	1 [Reference]		1 [Reference]	
25 000-50 000	1.91 (1.58-2.31)	<.001	1.39 (1.10-1.75)	.006
>50 000	2.34 (1.89-2.90)	<.001	1.36 (1.03-1.81)	.03
Cost barrier to care				
Yes	1 [Reference]		1 [Reference]	
No	1.61 (1.23-2.10)	<.001	1.31 (0.87-1.98)	.20
Educational level				
No high school diploma or attendance	1 [Reference]		1 [Reference]	
High school diploma or GED certificate	1.88 (1.47-2.39)	<.001	1.46 (1.07-2.00)	.02
Some college	2.65 (2.09-3.36)	<.001	1.79 (1.32-2.43)	<.001
College degree	3.45 (2.74-4.34)	<.001	1.93 (1.40-2.66)	<.001
Marital status				
Married	1 [Reference]		1 [Reference]	
Divorced, widowed, or separated	0.56 (0.50-0.64)	<.001	0.82 (0.68-0.98)	.30
Unmarried partnership	0.98 (0.56-1.73)	>.90	1.31 (0.81-2.14)	.90
Never married	0.57 (0.44-0.74)	.08	0.78 (0.48-1.28)	.13
Smoking status				
Never smoker	1 [Reference]		1 [Reference]	
Every day	0.69 (0.53-0.90)	.006	0.95 (0.72-1.27)	.70
Some days	0.62 (0.43-0.90)	.01	0.79 (0.54-1.14)	.20
Employment status				
Employed	1 [Reference]		1 [Reference]	
Unemployed	0.58 (0.42-0.80)	.001	0.94 (0.58-1.52)	.80
Homemaker	0.71 (0.24-2.05)	.50	1.04 (0.32-3.37)	>.90
Student	0.41 (0.16-1.08)	.07	0.23 (0.04-1.22)	.08
Retired	0.87 (0.75-1.01)	.08	1.02 (0.81-1.30)	.80

Abbreviations: GED, General Educational Development; OR, odds ratio; PCP, primary care physician, PSA; prostate-specific antigen.

^a^ Race and ethnicity were collected from the 2020 Behavioral Risk Factor Surveillance System survey data.

^b^ Estimated by zip code; lower bounds were noninclusive.

Even when accounting for a potential interaction between age and clinician-led discussion of PSA testing advantages in a multivariable model, such discussion had an association with increased screening (OR, 11.30; 95% CI, 8.58-14.90; *P* < .001), whereas males in the 80-year-or-older group no longer had a significant inverse association with recent screening (OR, 0.79; 95% CI, 0.57-1.09; *P* = .20) (eTable 1 in Supplement 1). The interaction between age and discussion of PSA testing advantages in males 80 years or older was slightly lower (OR, 0.94; 95% CI, 0.84-0.99; *P* = .005) compared with the interaction in those aged 70 to 74 years. The interaction between discussion of PSA testing advantages and 75 to 79 years of age was not significantly higher or lower from its interaction with 70 to 74 years of age. Conversely, discussion of PSA testing disadvantages did not have a significantly higher interaction with ages 75 to 79 years (OR, 1.53; 95% CI, 0.99-2.37; *P* = .06) or 80 years or older (OR, 0.83; 95% CI, 0.54-1.28; *P* = .40) compared with its interaction with age 70 to 74 years (eTable 2 in Supplement 1).

### PSA Screening and Sociodemographic Factors

**Figure 3.**
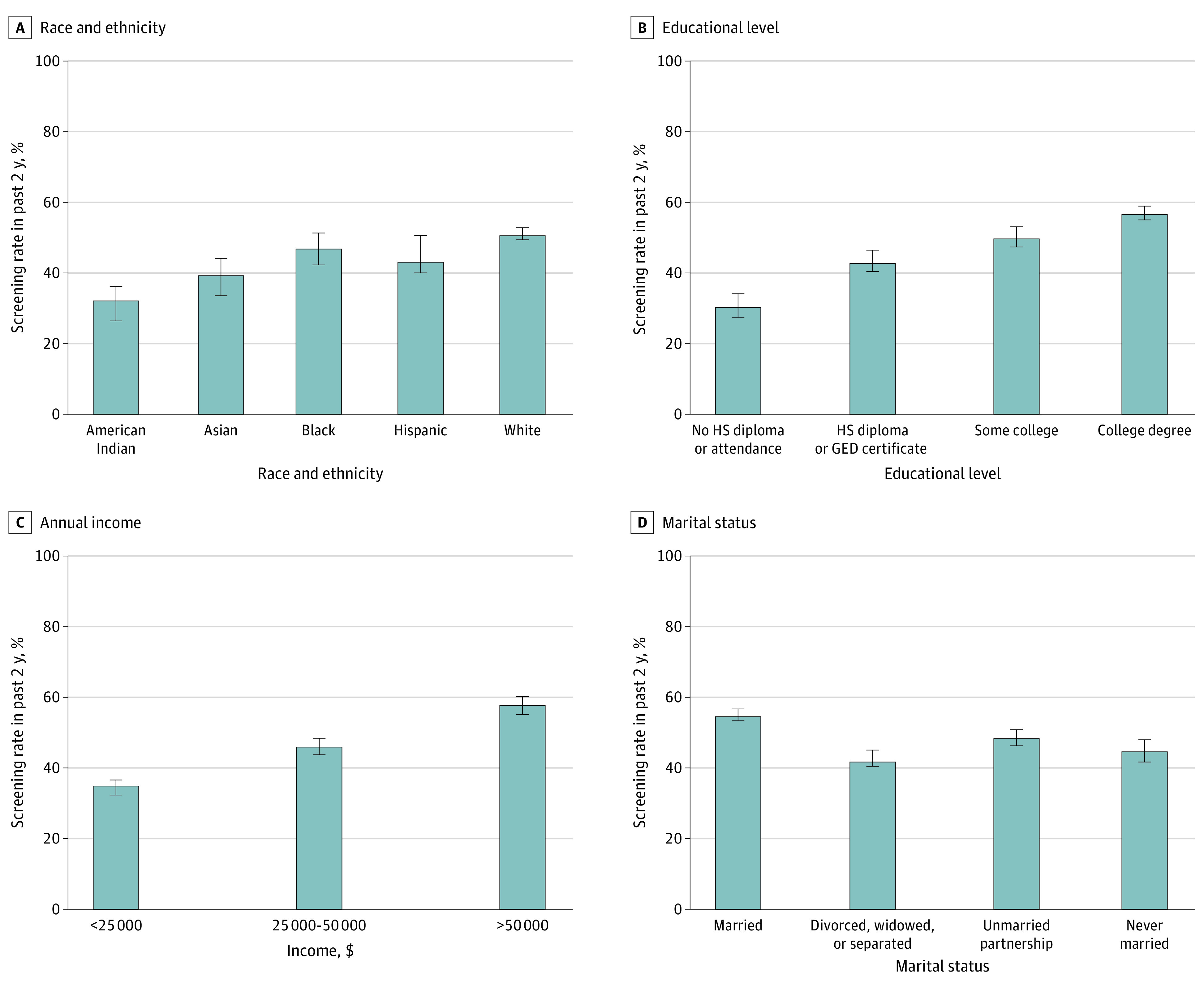
Screening Rates by Race and Ethnicity, Educational Level, Annual Income, and Marital Status Error bars represent 95% CIs. GED indicates General Educational Development; HS, high school.

Recent PSA screening rates were different by race and ethnicity (*P* < .001 from analysis of variance test): White males had the highest rate of 50.7% (95% CI, 49.9%-51.5%) and American Indian males had the lowest rate of 32.0% (95% CI, 26.2%-37.9%) (Figure 3A). Screening rates also differed by educational levels (*P* < .001): males with no high school diploma or attendance had the lowest rate of 30.3% (95% CI, 28.0%-32.7%), whereas those who had a college degree had the highest rate of 56.7% (95% CI, 55.3%-57.8%) (Figure 3B). Similarly, recent screening rates differed significantly and increased with higher annual income brackets (Figure 3C). Screening rates were also different by marital status (*P* < .001): married males had the highest rate of 54.4% (95% CI, 53.4%-55.4%), followed by those in an unmarried partnership (48.2%; 95% CI, 41%-55.4%), and those who were divorced, widowed, or separated had the lowest rate of 41.5% (95% CI, 40.2%-42.8%) (Figure 3D).

In a multivariable regression model, race and ethnicity were not associated with recent PSA screening. Compared with an annual income less than $25 000, an annual income more than $25 000 was associated with recent screening. Specifically, annual incomes of $25 000 to $50 000 (noninclusive lower bound) and more than $50 000 were associated with recent screening (OR, 1.39 [95% CI, 1.10-1.75; *P* = .006] and 1.36 [95% CI, 1.03-1.81, respectively; *P* = .03]). Compared with no high school diploma or attendance, a high school diploma or GED certificate (OR, 1.46; 95% CI, 1.07-2.00; *P* = .02), some college (OR, 1.79; 95% CI, 1.32-2.43; *P* < .001), or a college degree (OR, 1.93; 95% CI, 1.40-2.66; *P* < .001) were associated with recent screening. Compared with married males, those who were divorced, widowed, or separated were less likely to be recently screened (OR, 0.82; 95% CI, 0.68-0.98; *P* = .30). Having a cost barrier to care, employment status, and smoking status were not associated with recent screening. In the sensitivity analysis using unweighted survey data, we found no significant changes in results.

## Discussion

In this cohort of male respondents aged 70 years or older, recent PSA screening rates were high. Although the rate of screening decreased with age, even in the cohort of respondents 80 years or older, 39.4% had a PSA test within the past 2 years. We identified having a PCP, a discussion of PSA testing advantages with any clinician, a post–high school educational level, and an annual income more than $25 000 as factors associated with increased PSA screening among older males. We expect these findings to be important in focusing interventions for reducing low-value screening.

While discussion of the advantages of PSA testing had an association with increased screening, discussion of the disadvantages of PSA testing had no association with reduced screening, suggesting that any discussion of PSA testing likely occurs in the context of a clinician- or patient-initiated effort to ultimately screen for prostate cancer. There was an interaction, albeit with minimal absolute effect size, between age and discussing PSA testing advantages with a clinician, suggesting that such a discussion is slightly modified by age and associated with a higher likelihood of screening in relatively younger males.

Previously observed screening use patterns in younger age groups persist in the present cohort of older males. Specifically, high annual income and high educational level are well-established in the literature as factors in the use of PSA screening services,^10,11^ and we found similar increased screening use among individuals in higher annual income brackets and with higher levels of education. American Indian males had the lowest screening rate; while there were few patients in this racial group to power this analysis, this pattern was consistent with findings from other studies analyzing race and ethnicity–based PSA screening that reported underutilization of screening among American Indian males.^12^ The findings of this study were also consistent with existing literature that reported marriage as a factor in PSA screening use,^13,14,15^ as the divorced, widowed, or separated males in the present cohort had a significantly lower odds of recent screening compared with married males.

The practice of PSA screening in older males or those who are unlikely to benefit from screening predates the most recent iterations of the USPSTF guidelines.^6^ Prior work by Merrill et al^16^ that studied age-based PSA screening using the 2018 BRFSS survey data found a screening rate of 52.6% among those aged 70 to 79 years, which was the highest screening rate across all age groups in their study and closely matched the screening rate observed in the present study. Additionally, discussions of PSA testing advantages and disadvantages as well as annual income and educational level in this study were similar to the outcomes reported in the study by Merrill et al.^16^ Using veteran patient data from 2003, Walter et al^22^ reported a high PSA screening rate among older males, and when analyzing screening rates in the context of overall health, they found that screening did not substantially decline among those in poor health or those with several preexisting diseases. Given the substantial risks associated with PSA screening, older males, especially those in poor health, were more likely to experience the adverse consequences of PSA testing rather than any clinical benefit.^22^ Overall, the literature and findings from the present study suggest a strong practice inertia of low-value PSA screening and patient educational level among older males.

### Limitations

We recognize several limitations in this study. First, the BRFSS survey data divided respondents by 5-year age intervals without providing exact ages. Since national guidelines recommend PSA screening for males aged 55 to 69 years and we defined recent screening as PSA testing within the past 2 years, the 70-year-or-older age group in this study could possibly include males who were screened at age 68 or 69 years, which is within the recommended screening range. To assess the degree to which the 70-to-74-year age group captured males who were screened at 68 years of age, we measured the proportion of those in the 70-to-74-year age group who were screened within the past year. Of those in this age group, 81.3% were screened within the past year, indicating that the youngest possible age at time of screening was more likely to be 69 years rather than 68 years. While we recognize that a small percentage of the 70-year-or-older age group may include males aged 68 to 69 years, since 69 years is the oldest recommended age for PSA screening according to USPSTF guidelines,^6^ we believe the data analysis is still meaningful in the context of low-value PSA screening for older males who would likely not benefit from PSA screening.

Second, the BRFSS survey question of time since the last PSA testing did not distinguish between a PSA test for prostate cancer screening and a PSA test for prostate cancer monitoring or symptomatic noncancer prostate diseases, such as prostatitis or benign prostatic hyperplastia.^23^ We attempted to account for this gap by filtering out respondents who have had a past or current prostate cancer diagnosis, but we were unable to parse out which respondents also had other prostate diseases that would prompt routine and longitudinal PSA monitoring. We also could not distinguish which guidelines physicians were using to administer PSA tests, as some guidelines (those from the American Cancer Society and American Urological Association) suggest considering life expectancy when screening males who are older than 70 years.^24,25^ According to such guidelines, some of the older males who had PSA screening in this study may be target recipients if they had longer life expectancies, but the nature of the BRFSS database did not allow us to assess respondent life expectancy. It is also important to note that the survey question of whether a clinician discussed the advantages and/or disadvantages of PSA testing did not have an associated time component; thus, these discussions could have taken place at any time before the PSA test. Knowing the timeframe of these discussions in relation to timing of recent PSA testing could have provided further context to the reasons for the PSA screening.

Third, the 2020 BRFSS survey had a response rate of 47.9%, and some states did not offer the prostate cancer module associated with this survey,^26^ potentially introducing selection bias and limited generalizability. This issue is further compounded by the PSA screening module being optional in the survey, potentially creating sampling bias. However, in using the BRFSS data–provided weights in the statistical analysis, we aimed to maximize generalizability to the national population. Given that the BRFSS survey is a cross-sectional survey administered over the telephone, poor recall or response bias among respondents could also be an issue.^19^

## Conclusions

In this survey study of PSA screening using national data, older males appeared to be overscreened for prostate cancer, despite recent changes in USPSTF guidelines^6^ that established an explicit age cutoff for PSA screening. Clinician-led discussion of PSA test advantages was associated with increased PSA screening regardless of whether survey respondents reported a discussion of the disadvantages of PSA testing, suggesting that changes could be made at the clinician level to increase adherence to age-based PSA testing guidelines. The findings also highlighted that older males with regular contact with a PCP, higher educational level, and higher annual income were more likely to be recipients of low-value PSA screening. The results of this study can inform interventions to disincentivize low-value screening. For instance, given the higher educational levels among respondents who may overuse PSA screening, direct patient education on the risks of PSA screening may be beneficial for males older than 70 years. Future work should quantify the implications of this screening, specifically the consequences of overtreatment for patients.

## Notes

Supplement 1. **eTable 1.** Multivariable Logistic Regression Assessing Odds of Recent Screening With an Interaction Term Between Age and Provider-Led Discussion of PSA Advantages **eTable 2.** Multivariable Logistic Regression Assessing Odds of Recent Screening With an Interaction Term Between Age and Provider-Led Discussion of PSA Disadvantages

Supplement 2.
Data Sharing Statement
