# Supplementary material for: Low-Value Prostate-Specific Antigen Screening in Older Males
Source: JAMA Netw Open. 2023 Apr 11;6(4):e237504. doi: 10.1001/jamanetworkopen.2023.7504 (PMC10091155; doi:10.1001/jamanetworkopen.2023.7504)
Supplement: Supplement 1. — eTable 1. Multivariable Logistic Regression Assessing Odds of Recent Screening With an Interaction Term Between Age and Provider-Led Discussion of PSA Advantages eTable 2. Multivariable Logistic Regression Assessing Odds of Recent Screening With an Interaction Term Between Age and Provider-Led Discussion of PSA Disadvantages [file jamanetwopen-e237504-s001.pdf]

Supplemental Online Content

Kalavacherla S, Riviere P, Javier-Desloges J, et al. Low-value prostate-specific antigen screening in older males. *JAMA Netw Open*. 2023;6(4):e237504.
doi:10.1001/jamanetworkopen.2023.7504

eTable 1. Multivariable Logistic Regression Assessing Odds of Recent Screening With an Interaction Term Between Age and Provider-Led Discussion of PSA Advantages

eTable 2. Multivariable Logistic Regression Assessing Odds of Recent Screening With an Interaction Term Between Age and Provider-Led Discussion of PSA Disadvantages

This supplemental material has been provided by the authors to give readers additional information about their work.

© 2023 Kalavacherla S et al. *JAMA Network Open.*

eTable 1. Multivariable logistic regression assessing odds of recent screening with an interaction term between age and provider-led discussion of PSA advantages.

Abbreviations: PCP = primary care physician, PSA = Prostate-specific antigen, HS = high school

Characteristic	OR (95% CI) [1]	p-value
**Age**		
70-74	—	
75-79	0.94 (0.68, 1.30)	0.7
≥80	0.79 (0.57, 1.09)	0.2
**Have a PCP**		
No	—	
Yes	1.89 (1.41, 2.54)	<0.001
**Discussed PSA advantages with a healthcare provider**		
No	—	
Yes	11.3 (8.58, 14.9)	<0.001
**Discussed PSA disadvantages with a healthcare provider**		
No	—	
Yes	0.95 (0.77, 1.17)	0.6
**Race**		
Non-Hispanic White	—	
Non-Hispanic Black	0.87 (0.64, 1.20)	0.4
Non-Hispanic Asian	0.72 (0.35, 1.51)	0.4
Non-Hispanic Native American	0.58 (0.32, 1.05)	0.072
Hispanic	1.29 (0.82, 2.03)	0.3
**Income (as estimated by zip code; non-inclusive lower bounds)**		
$0-25k	—	
$25k-50k	1.39 (1.10, 1.76)	0.005
>$50k+	1.03 (0.77, 1.36)	0.9
**Cost barrier to care**		
Yes	—	
No	1.33 (0.88, 2.01)	0.2
**Highest level of education completed**		
No HS	—	
HS general equivalence degree	1.48 (1.09, 2.01)	0.013
Some college	1.8 (1.33, 2.44)	<0.001
College	1.93 (1.41, 2.65)	<0.001
**Marital status**		
Married	—	
Divorced/widowed/separated	0.82 (0.68, 0.98)	0.031
Partnership	1.32 (0.81, 2.15)	0.3
Never married	0.78 (0.47, 1.29)	0.3
**Smoking status**		
Never smoker	—	
Everyday	0.95 (0.71, 1.27)	0.7
Some days	0.79 (0.54, 1.15)	0.2
**Employment status**		
Employed	—	
Unemployed	0.93 (0.57, 1.51)	0.8
Homemaker	1 (0.31, 3.21)	>0.9
Student	0.21 (0.04, 1.10)	0.065
Retired	1.02 (0.80, 1.30)	0.9
**Interaction: Ages * Discussion of PSA advantages**		
70-74 * yes	—	
75-79 * yes	0.86 (0.57, 1.30)	0.5
≥80 * yes	0.94 (0.84, 0.99)	0.005

[1] OR = Odds Ratio, CI = Confidence Interval

© 2023 Kalavacherla S et al. *JAMA Network Open.*

eTable 2. Multivariable logistic regression assessing odds of recent screening with an interaction term between age and provider-led discussion of PSA disadvantages.

Abbreviations: PCP = primary care physician, PSA = Prostate-specific antigen, HS = high school

Characteristic		OR (95% CI) [1]	p-value
**Age**			
	70-74	—	
	75-79	0.75 (0.60, 0.95)	0.016
	≥80	0.56 (0.44, 0.72)	<0.001
**Have a PCP**			
	No	—	
	Yes	1.89 (1.41, 2.54)	<0.001
**Discussed PSA advantages with a healthcare provider**			
	No		
	Yes	9.32 (7.64, 11.4)	<0.001
**Discussed PSA disadvantages with a healthcare provider**			
	No	—	
	Yes	0.89 (0.68, 1.15)	0.4
**Race**			
	Non-Hispanic White	—	
	Non-Hispanic Black	0.87 (0.64, 1.18)	0.4
	Non-Hispanic Asian	0.71 (0.34, 1.48)	0.4
	Non-Hispanic Native American	0.58 (0.32, 1.05)	0.074
	Hispanic	1.28 (0.82, 1.99)	0.3
**Income (as estimated by zip code; non-inclusive lower bounds)**			
	$0-25k	—	
	$25k-50k	1.41 (1.12, 1.77)	0.004
	>$50k+	1.04 (0.78, 1.38)	0.8
**Cost barrier to care**		1.25 (0.94, 1.66)	
	Yes	—	
	No	1.31 (0.87, 1.97)	0.2
**Highest level of education completed**			0.11
	No HS	—	
	HS general equivalence degree	1.46 (1.07, 1.98)	
	Some college	1.78 (1.31, 2.40)	0.016
	College	1.93 (1.41, 2.64)	<0.001
**Marital status**			<0.001
	Married	—	
	Divorced/widowed/separated	0.82 (0.68, 0.98)	
	Partnership	1.31 (0.80, 2.14)	0.031
	Never married	0.77 (0.47, 1.28)	0.3
**Smoking status**			0.3
	Never smoker	—	
	Everyday	0.96 (0.72, 1.28)	
	Some days	0.79 (0.54, 1.14)	0.8
**Employment status**			0.2
	Employed	—	
	Unemployed	0.96 (0.60, 1.55)	
	Homemaker	1.01 (0.32, 3.17)	0.9
	Student	0.24 (0.04, 1.30)	>0.9
	Retired	1.02 (0.81, 1.30)	0.1
**Interaction: Ages * Discussion of PSA disadvantages**			
	70-74 * yes	—	
	75-79 * yes	1.53 (0.99, 2.37)	0.055
	≥80 * yes	0.83 (0.54, 1.28)	0.4

[1] OR = Odds Ratio, CI = Confidence Interval

© 2023 Kalavacherla S et al. *JAMA Network Open.*
