# Supplementary material for: Low-Value Prostate-Specific Antigen Screening in Older Males
Source: JAMA Netw Open. 2023 Apr 11;6(4):e237504. doi: 10.1001/jamanetworkopen.2023.7504 (PMC10091155; doi:10.1001/jamanetworkopen.2023.7504)
Supplement: Supplement 2. — Data Sharing Statement [file jamanetwopen-e237504-s002.pdf]

Data Sharing Statement

Kalavacherla. Low-Value Prostate-Specific Antigen Screening in Older Males. *JAMA Netw Open*. Published April 11, 2023. doi:10.1001/jamanetworkopen.2023.7504

Data
Data available: No

Additional Information
Explanation for why data not available: The survey data used in this study is already publicly available on the Centers for Disease Control (CDC) website: https://www.cdc.gov/brfss/annual_data/annual_2020.html
